# Physical Match Demands of International Women’s Rugby Union: A Three-Year Longitudinal Analysis of a Team Competing in The Women’s Six Nations Championship

**DOI:** 10.3390/jfmk8010032

**Published:** 2023-03-02

**Authors:** David Nolan, Orlaith Curran, Aidan J. Brady, Brendan Egan

**Affiliations:** 1 School of Health and Human Performance, Dublin City University, D09 V209 Dublin, Ireland; 2 Irish Rugby Football Union, D04 F720 Dublin, Ireland; 3 Insight Centre for Data Analytics, Dublin City University, D09 V209 Dublin, Ireland; 4 Florida Institute for Human and Machine Cognition, Pensacola, FL 32502, USA

**Keywords:** conditioning, female athlete, running, team sport

## Abstract

There is a paucity of studies describing the physical match demands of elite international women’s rugby union, which limits coaches’ ability to effectively prepare players for the physical demands required to compete at the elite level. Global positioning system technologies were used to measure the physical match demands of 53 international female rugby union players during three consecutive Women’s Six Nations Championships (2020–2022), resulting in 260 individual match performances. Mixed-linear modelling was used to investigate differences in physical match demands between positions. Significant effects (*p* < 0.05) of the position were observed for all variables, with the exception of relative distances (m.min^−1^) at velocities of 1.01–3.00 m·s^−1^ (*p* = 0.094) and 3.01–5.00 m·s^−1^ (*p* = 0.216). This study provides valuable data on the physical match demands of elite international women’s rugby union match play that may aid practitioners in the physical preparation of players to compete at this level. Training methodologies for elite-level female rugby union players should consider the unique demands across positional groups with specific considerations of high-velocity running and collision frequency.

## 1. Introduction

The women’s rugby union has experienced significant growth in participation numbers in recent years, with approximately 1 million active players registered globally in 2021 [1]. Despite this growth and the professionalization of the male game that began almost three decades ago, the majority of “elite” (international-level) female players in the modern era have participated as amateurs [2]. The Women’s Six Nations, existing in its current format since 2002, is an annual international rugby union competition contested among six top-ranked European teams (UK, France, Ireland, Italy, Scotland, and Wales) [3]. From 2016 onwards, the decision by specific nations to provide professional contracts to their international players has resulted in the Women’s Six Nations Championship simultaneously consisting of professional, semiprofessional, and amateur teams.

Rugby union can be described as an invasion field-based team sport consisting of intermittent bouts of high-intensity efforts (i.e., running, sprinting, tackling, rucking, mauling, and scrummaging) and periods of lower-intensity activity (i.e., walking, jogging, and resting) [4]. The improved sophistication and increased use of global positioning systems (GPS) technology in rugby union has enabled the in-depth analysis of the physical demands of match play [5,6], i.e., running distances, velocities, accelerations, decelerations, and related variables. Modern GPS technology also provides valid measures of collision events in rugby union [7]. These data may provide practitioners with useful metrics regarding physical match demands and monitoring training load, thereby aiding in the design of appropriate physical conditioning programs to improve tolerance to the demands of match play, potentially improving performance and reducing injury risk [8,9]. The match demands of men’s rugby union are well-documented across both playing level [4,10,11] and age grades [12]. A paucity of studies describing match demands of women’s rugby union exists [13], particularly at the international level, with previous studies using lower-ranked teams and small samples [14,15]. The most comprehensive analysis of the physical demands of women’s international rugby union to date reported differences between playing positions in high-velocity running (>5.5 m·s^−1^), accelerations, decelerations, and collisions [16]. Notably, this cohort ranked in the top 2 teams globally for the entirety of the data collection period, with several years consisting of professional players. The inclusion of data from professional players may limit the extrapolation of these findings to the wider international women’s rugby union population, as the majority of players are amateur. The heterogeneity of the methodology employed in previous studies of international cohorts [14,15,16], specifically the inclusion/exclusion criteria for match files and the used velocity thresholds, limit further comparison and synthesis of the existing literature.

This study provides the three-year longitudinal analysis of physical match demands of an elite, amateur, women’s international rugby union team competing in the Women’s Six Nations Championship. The study also investigates the influence of playing position, which was hypothesized to have a significant effect on physical match demands.

## 2. Materials and Methods

Following institutional ethical approval (REF: DCUREC/2022/012), a three-year longitudinal analysis of the physical match demands of the Women’s Six Nations Championship was conducted between 2020 and 2022 inclusive. A total of 53 players from a single team generated 260 match files from 12 matches (mean individual match files: 3.9 ± 2.6; median: 3; range: 1–12) across the three successive campaigns. Individual positions were categorized into seven groups: front-row (FR; n = 11, prop and hooker), second-row (2R, n = 7), back-row (BR; n = 9, flanker and number eight), scrum-half (SH, n = 5), fly-half (FH, n = 5), center (C, n = 5), back-three (B3; n = 11, winger and full-back).

All matches took place between the hours of 12:00 and 22:00. Physical match demands were quantified using APEX GPS units (STATSports Apex; STATSports, Newry, Ireland), which were switched on and fitted at least 15 min prior to the start of match play which is recommended to improve connectivity [17]. These devices demonstrate typical measurement error of <5% in coefficient of variation (CV), with close (<2% CV) comparisons to sport-specific variable measurements i.e., distance covered and peak velocity [18,19]. Files were downloaded into the manufacturer’s software for analysis, with warm-up and half-time periods removed post hoc. All GPS files were included in the analysis, regardless of time on pitch (mean: 69.23 ± 32.49 min; median: 77.97 min; range: 1.75–123.48 min).

The analyzed variables were: total distance, distances covered at <1.00, 1.01–3, 3.01–5.00, 5.01–5.50, and >5.50 m·s^−1^. Total collisions (contacts >8 g) were also recorded. Maximal velocity was determined via a 40 m sprint test conducted in the preseason periods using the SmartSpeed single-beam timing gates system (VALD, Brisbane, Australia). Average velocity achieved from the 30–40 m split was computed as the maximal velocity and manually imported to the manufacturer’s software. Maximal velocity was automatically increased by the manufacturer’s software in the event of an individual achieving velocities greater than those assigned on three subsequent occasions during match play, with the average of these three higher velocities becoming the updated maximal velocity. Acceleration and deceleration metrics were recorded, but not included in the final analysis due to high levels of error for these specific metrics [20]. All variables are expressed in absolute terms (m) and relative to playing time (m.min^−1^). Arbitrary thresholds were set to align with, and allow for comparison, where possible, to previous studies on female rugby [14,15,16,21,22].

Statistical analysis was completed using the Statistical Package for the Social Sciences SPSS v.27, IBM, Chicago, IL, USA). Data are presented as mean ± standard deviation (SD) unless otherwise stated. All variables were log transformed prior to statistical analysis. Differences in the relative physical demands between playing positions were examined using a series of linear mixed models. Playing seasons (2020, 2021, and 2022) and position were treated as the fixed effects, and individual players were treated as a random effect. Significant fixed effects were probed using post hoc Bonferroni comparisons. Due to variations in minutes played across positions and matches, statistical analysis was only performed on data for relative to playing time (m.min^−1^), but not absolute distances (m). All statistical analyses accepted significance at *p* < 0.05.

## 3. Results

Physical match demands are reported in Table 1, Table 2 and Table 3. The pairwise comparisons of relative physical match demands separated by playing position are shown in Table 3. Significant effects of the position were observed for all variables, with the exception of relative distances at velocities of 1.01–3.00 and 3.01–5.00 m·s^−1^.

## 4. Discussion

This study provides valuable descriptive data regarding the physical match demands of international women’s rugby union while also investigating the influence of playing position on relative demands of match play. Similar to previous studies [15,16,21], playing position had a significant effect on physical match demands in the present study, with the exception of relative distances at velocities of 1.01–3.00 and 3.01–5.00 m·s^−1^. Total distance separated by position in the present study was 3232–5173 m, which is similar to previously reported top-ranked international female rugby union players (3240–5283 m) [16], but lower than the 5784 and 5820 m reported in other international cohorts [14,15]. This distance is also lower than the 4982 m reported for English premiership players [22]. The higher absolute values reported for other international cohorts were likely due to the decision to only include match files from players who took part in ≥60 min of match play [15,22] or complete match files [14] compared to the present study, which included all match files regardless of playing time. Relative distance for the present cohort (59.6 m.min^−1^) was lower than that previously reported in international and club-level female rugby (65.9–68.3 m.min^−1^) [14,16,21]. Given that peak velocity achieved in the present study (6.8 m.min^−1^) was higher than that previously reported (6.1 m.min^−1^) [14], and collision frequency (0.46 collisions per min) was also higher than that previously reported [16], the lower relative distance in the present study may have been due to contextual match constraints, i.e., technical and tactical factors.

Total distance covered at > 5.5 m·s^−1^ accounted for ~2.5% of the total distance, which was higher than the ~1.2% previously reported in a similarly ranked cohort [14], but similar to the ~2.7% reported for a top-ranked team [16]. Distance covered at high velocities, rather than the absolute distance covered during match play was posited as a differentiating factor between top- and lower-ranked teams [23]. This has not yet been found in female rugby union and may warrant further investigation. Due to the similarities observed in the physical match demands of the current cohort to those of a top-ranked professional team, it may be that factors other than physical match demands are the key differentiating factors between top- and lower-ranked teams, i.e., technical and tactical ability.

Akin to previous reports [15,16], FR and SH covered the least total distances, but similar to these studies, this observation can be attributed to substitution strategy, with FR and SH playing fewer minutes than those in other positions. FR displayed lower relative distances and peak velocities than those of backs (C and B3), which is consistent with that previously reported in international cohorts [16]. Relative running demands are similar between the back positional groups, with no differences found apart from SH displaying lower peak velocity achieved in match play than that in B3. Similarly, no differences were found between the different forward positions (FR, 2R, and BR) for relative demands. The current cohort displayed a higher homogeneity of relative demands across positional groups (forwards and backs) than that previously reported [15,16], but this may be attributable to the larger number of match files used in those studies.

Collision frequency by position (ranging from 0.23 to 0.89 collisions per min) in the present study was higher than that previously reported in women’s international rugby union (0.17 to 0.33 collisions per min) [16] and in male rugby union (0.18 to 0.44 collisions per min) [7]. These differences may have been the result of specific tactical approaches adopted by teams during these specific matches. Women’s rugby union has adopted more possession-driven attacking tactics compared to increased kicking frequency in the male game [24]. This open and continuous style of play in the female game may explain the increased collision frequency. There is also a large difference in sample size and number of matches captured in the present study compared to previous analysis of women’s international rugby union (n = 260, 12 matches vs. n = 967, 53 matches) [16]. This likely resulted in the latter cohort being exposed to a wider range of teams of varying tactical strategy and technical ability, which may explain the differences in collision frequency reported.

An important consideration is that all data presented in the present study were collected across the 2020, 2021, and 2022 championships, which coincided with the COVID-19 global pandemic. The pandemic caused a significant disruption to the normal functioning of the Women’s Six Nations Championship including delays, the rescheduling of matches, the cancellation of matches, and the restriction of spectator attendance. COVID-19 restrictions and public health policies may have also impacted training scheduling and player availability.

There are several limitations to this study; however, they are not unique to this investigation, but rather characteristic of this research area. The present study utilized data from only one team, which may have limited its application to other elite teams given the complex and contextual nature of match demands [25]. Inconsistency in methodological approaches across studies specifically regarding inclusion/exclusion criteria and application of velocity thresholds hinders direct comparison. Classification issues due to a lack of consensus regarding velocity thresholds may be compounded further in female team sports due to limited research [26]. The reported data describe the physical match demands, but do not account for the influence of differing tactical strategies adopted by the team and/or their opposition; thus, these data should be interpreted with an appreciation of relevant contextual factors, e.g., weather conditions, the level of opposition, playing style, and match significance. Ball-in-play data were not collected in the present study, which could significantly influence the relative intensity and collision frequency [27]. Practitioners should, therefore, be cognizant of potential contextual factors when interpreting the data provided in this study.

Since the completion of this investigation, it has been announced that the international team, which is the focus of the present study, intend to offer a number of professional contracts to players, effectively transitioning into a semiprofessional environment [28]. All other amateur teams competing in the Women’s Six Nations Championship have recently announced similar intentions [29,30,31]; thus, the 2023 Women’s Six Nations Championship, for the first time, will consist of only professional and semiprofessional teams rather than also including amateur teams. Future research should investigate factors other than physical match demands that differentiate high- and lower-ranked teams.

## 5. Conclusions

The present study provided the descriptive data of the physical demands of match play from an elite, amateur, women’s international rugby union team competing in the Women’s Six Nations Championship across a three-year period. Total running demands were similar to those previously reported in other international cohorts employing similar methodology, but the number of collisions per minute was higher than that previously reported in similar cohorts. Differences in the physical demands were found between positions, and practitioners should be aware of the broad demands of match play and position-specific differences when designing training programs. Training methodologies for elite-level female rugby union players should consider the unique demands across positional groups with specific consideration of high-velocity running and collision frequency.

## Figures and Tables

**Table 1:** Physical match demands of match play for a squad of international female rugby union players during three consecutive years of the Women’s Six Nations Championship.

	Mean
Time played	69.2 ± 32.5
Total distance (m)	4177 ± 2066
Relative distance (m.min^−1^)	59.6 ± 8.68
Peak velocity achieved (m·s^−1^)	6.76 ± 0.97
Percentage of maximum velocity achieved (%)	85.7 ± 8.95
Total distance < 1 m·s^−1^ (m)	741 ± 378
Relative distance < 1 m·s^−1^ (m.min^−1^)	10.6 ± 1.70
Total distance at 1.01–3.00 m·s^−1^ (m)	2076 ± 1036
Relative distance at 1.01–3.00 m·s^−1^ (m.min^−1^)	29.5 ± 4.53
Total distance at 3.01–5.00 m·s^−1^ (m)	1157 ± 637
Relative distance at 3.01–5.00 m·s^−1^ (m.min^−1^)	16.6 ± 5.80
Total distance at 5.01–5.50 m·s^−1^ (m)	97.0 ± 75.8
Relative distance at 5.01–5.50 m·s^−1^ (m.min^−1^)	1.40 ± 0.89
Total distance at > 5.50 m·s^−1^ (m)	106 ± 126
Relative distance at > 5.50 m·s^−1^ (m.min^−1^)	1.51 ± 1.71
Total collisions (n)	31.6 ± 39.3
Collisions per min	0.46 ± 0.48

**Table 2:** Positional differences in the *absolute* physical match demands of match play for a squad of international female rugby union players during three consecutive years of the Women’s Six Nations Championship.

	FR (n = 62)	2R (n = 35)	BR (n = 47)	SH (n = 28)	FH (n = 13)	C (n = 28)	B3 (n = 47)
Time played (min)	58.1 ± 31.8	74.7 ± 28.6	73.2 ± 33.2	56.2 ± 33.3	63.6 ± 33.9	77.8 ± 35.2	80.2 ± 27.0
Total distance (m)	3232 ± 1812	4490 ± 1779	4118 ± 1967	3541 ± 2203	4231 ± 2362	4917 ± 2219	5173 ± 1842
Total distance < 1 m·s^−1^ (m)	542.0 ± 294	731 ± 282	849 ± 414	564 ± 350	725 ± 392	887 ± 408	925 ± 345
Total distance at 1.01–3.00 m·s^−1^ (m)	1740 ± 987	2197 ± 905	2086 ± 1045	1709 ± 1108	2032 ± 1095	2434 ± 1114	2438 ± 912
Total distance at 3.01–5.00 m·s^−1^ (m)	863 ± 579	1432 ± 619	1057 ± 510	1100 ± 754	1213 ± 759	1306 ± 614	1367 ± 588
Total distance at 5.01–5.50 m·s^−1^ (m)	50.3 ± 54.7	80.4 ± 64.0	75.3 ± 50.0	92.4 ± 73.1	136 ± 91.7	133 ± 68.0	163 ± 74.3
Total distance at > 5.50 m·s^−1^ (m)	36.1 ± 57.6	49.8 ± 48.8	49.7 ± 44.5	75.5 ± 73.2	125 ± 96.7	158 ± 118	280 ± 149
Total collisions (n)	24.7 ± 29.2	24.0 ± 28.1	67.6 ± 56.0	20.1 ± 29.6	17.2 ± 25.7	26.3 ± 29.5	24.2 ± 31.8

FR = front row, 2R = second row, BR = back row, SH = scrum half, FH = fly half, C = center, B3 = back three.

**Table 3:** Positional differences with pairwise comparisons in the *relative* physical match demands of match play for a squad of international female rugby union players during three consecutive years of the Women’s Six Nations Championship.

	FR (n = 62)	2R (n = 35)	BR (n = 47)	SH (n = 28)	FH (n = 13)	C (n = 28)	B3 (n = 47)
Relative distance (m.min^−1^)	55.0 ± 7.58 ^g^	59.4 ± 5.55	56.3 ± 8.63 ^g^	61.3 ± 11.9	64.5 ± 7.31	63.5 ± 4.52	64.4 ± 7.76 ^a,c^
Peak velocity achieved (m·s^−1^)	6.14 ± 0.77 ^f,g^	6.32 ± 0.54 ^g^	6.51 ± 0.96 ^g^	6.56 ± 0.71 ^g^	6.95 ± 0.68	7.27 ± 0.81 ^a^	7.92 ± 0.56 ^a,b,c,d^
Percentage of maximum velocity achieved (%)	82.6 ± 8.18 ^f,g^	83.9 ± 6.60 ^g^	82.9 ± 11.4 ^g^	84.8 ± 9.22	86.8 ± 7.97	90.2 ± 7.10 ^a^	91.4 ± 5.77 ^a,b,c^
Relative distance < 1 m·s^−1^ (m.min^−1^)	9.47 ± 1.21 ^f,g^	9.85 ± 0.80	11.4 ± 1.94	10.2 ± 1.45	11.6 ± 1.19	11.6 ± 1.38 ^a^	11.5 ± 1.67 ^a^
Relative distance at 1.01–3.00 m·s^−1^ (m.min^−1^)	29.6 ± 4.63	29.0 ± 3.58	27.7 ± 5.18	29.0 ± 5.12	31.1 ± 3.88	31.4 ± 3.23	30.2 ± 4.21
Relative distance at 3.01–5.00 m·s^−1^ (m.min^−1^)	14.6 ± 5.39	18.7 ± 3.65	15.6 ± 7.92	18.9 ± 7.46	17.7 ± 5.38	16.9 ± 2.69	16.9 ± 4.48
Relative distance at 5.01–5.50 m·s^−1^ (m.min^−1^)	0.85 ± 0.81 ^d,e,f,g^	1.14 ± 0.92 ^e,g^	1.05 ± 0.52 ^f,g^	1.65 ± 0.95 ^a^	2.07 ± 0.62 ^a,b^	1.74 ± 0.63 ^a,c^	2.09 ± 0.73 ^a,b,c^
Relative distance at > 5.50 m·s^−1^ (m.min^−1^)	0.55 ± 0.76 ^e,f,g^	0.79 ± 0.89 ^g^	0.66 ± 0.62 ^g^	1.60 ± 1.67	2.01 ± 1.24 ^a^	1.92 ± 1.12 ^a^	3.73 ± 2.10 ^a,b,c^

FR = front row, 2R = second row, BR = back row, SH = scrum half, FH = fly half, C = center, B3 = back three. ^a, b, c, d, e, f, g^ significantly different from FR, 2R, BR, SH, FH, C, and B3, respectively.

## Data Availability

Data are available upon request.
